# Gene methylation biomarkers in sputum as a classifier for lung cancer risk

**DOI:** 10.18632/oncotarget.19255

**Published:** 2017-07-15

**Authors:** Shuguang Leng, Guodong Wu, Donna M. Klinge, Cynthia L. Thomas, Elia Casas, Maria A. Picchi, Christine A. Stidley, Sandra J. Lee, Seena Aisner, Jill M. Siegfried, Suresh Ramalingam, Fadlo R. Khuri, Daniel D. Karp, Steven A. Belinsky

**Affiliations:** ^1^ Lung Cancer Program, Lovelace Respiratory Research Institute, Albuquerque, NM, USA; ^2^ Department of Internal Medicine, University of New Mexico, Albuquerque, NM, USA; ^3^ Dana-Farber Cancer Institute, Harvard Medical School, Boston, MA, USA; ^4^ Rutgers New Jersey Medical School, Newark, NJ, USA; ^5^ Department of Pharmacology, University of Minnesota, Minneapolis, MN, USA; ^6^ Department of Hematology and Medical Oncology, Winship Cancer Institute of Emory University, Atlanta, GA, USA; ^7^ MD Anderson Cancer Center, Houston, TX, USA

**Keywords:** gene methylation, lung cancer risk, biomarker, CT screening

## Abstract

CT screening for lung cancer reduces mortality, but will cost Medicare ∼2 billion dollars due in part to high false positive rates. Molecular biomarkers could augment current risk stratification used to select smokers for screening. Gene methylation in sputum reflects lung field cancerization that remains in lung cancer patients post-resection. This population was used in conjunction with cancer-free smokers to evaluate classification accuracy of a validated eight-gene methylation panel in sputum for cancer risk. Sputum from resected lung cancer patients (n=487) and smokers from Lovelace (n=1380) and PLuSS (n=718) cohorts was studied for methylation of an 8-gene panel. Area under a receiver operating characteristic curve was calculated to assess the prediction performance in logistic regressions with different sets of variables. The prevalence for methylation of all genes was significantly increased in the ECOG-ACRIN patients compared to cancer-free smokers as evident by elevated odds ratios that ranged from 1.6 to 8.9. The gene methylation panel showed lung cancer prediction accuracy of 82–86% and with addition of clinical variables improved to 87–90%. With sensitivity at 95%, specificity increased from 25% to 54% comparing clinical variables alone to their inclusion with methylation. The addition of methylation biomarkers to clinical variables would reduce false positive screens by ruling out one-third of smokers eligible for CT screening and could increase cancer detection rates through expanding risk assessment criteria.

## INTRODUCTION

Lung cancer (LC) remains the leading cause of cancer-related death for men and women in the US [1]. The success of CT screening in the National Lung Screening trial (NLST) for reducing LC mortality led to the recommendation by The Centers for Medicare and Medicaid (CMS) to screen people ages 55 to 77 who have a minimum 30 pack-year smoking history and currently smoke or have quit within the past 15 years [2]. However, these eligibility criteria or similar criteria by NCCN only capture 40% of the incident LC cases [3, 4]. Screening is estimated to save more than 12,000 lives, but cost Medicare ∼2 billion dollars, annually [5, 6]. This is due in part to the high false positive rate of CT screening as evident by the 39% of NLST participants that had at least one positive screening result (detection of indeterminate nodule) with >96% of those findings being classified as false positive [7].

The addition of molecular biomarkers interrogated in accessible biologic fluids such as sputum could provide better risk stratification to prioritize selection of smokers for CT screening and thereby substantially improve its predictive value and lower costs by reducing follow-up screens and biopsies [8, 9]. Gene silencing through methylation of cytosine in CpG islands in conjunction with chromatin remodeling leads to the development of heterochromatin of the gene promoter region, which denies access to regulatory proteins needed for transcription [10]. This epigenetically driven process is a major and causal event silencing hundreds of genes involved in all aspects of normal cellular function during LC initiation and progression [10]. Others and we have shown that gene specific promoter hypermethylation detected in sputum provides an assessment of field cancerization within the lungs of smokers that in turn predicts LC [11–17]. Specifically, our group showed that detecting gene methylation in exfoliated cells could predict cancer up to 18 months prior to clinical diagnosis, and was independently validated through case-control studies for predicting LC risk [11, 12]. However, the incorporation of this validated methylation panel in sputum into existing risk assessment models has not been assessed in a population-based setting that could identify high-risk smokers who would benefit most from a CT screen. A major challenge in conducting a prospective study for predicting LC risk is the need for a large population of high-risk smokers to yield enough cases of LC to accurately define the performance of the methylation panel.

Our previous case-control study used prevalent Stage I LC patients compared to cancer-free smoker controls to validate gene methylation panels for predicting LC [12]. Prior findings support our hypothesis of an expanding field of precancerous changes throughout the aerodigestive tract demonstrated initially through histologic changes and subsequently by increasing frequencies of genetic and epigenetic changes detected in exfoliated cells as the cancer develops [11, 16, 18]. Thus, the increase in number of cancer-associated methylated genes, rather than a single gene, is used in risk prediction [12]. The current study addressed whether our validated gene methylation panel could be extended to improve the existing risk prediction model used to recommend people for a CT screen. To accomplish this goal we used three cohorts of people: ECOG-ACRIN5597 trial participants who had a confirmed Stage I diagnosis of LC (based on pathology following surgical resection), the Lovelace Smokers Cohort ([LSC], current and former smokers at high risk for LC), and the PLuSS Smokers cohort (also current and former smokers at high risk for LC). The ECOG-ACRIN5597 participants were recruited from within the U.S. and Canada to participate in a prevention trial using L-selenomethione [19]. Patients had undergone surgical resection prior to trial enrollment and baseline sputum was obtained prior to randomization to the placebo or intervention group. Gene methylation in sputum reflects lung field cancerization that remains in lung cancer patients post-resection [20]. We hypothesized that because our gene methylation test is based on detecting the field of injury in the lung and not the actual small tumor present, the ECOG-ACRIN5597 trial participants would still have extensive field cancerization and serve in our study as people who should receive a CT screen, while the smokers selected were all cancer-free at time of sputum collection. We initially evaluated the utility of the eight gene panel to classify risk for LC by comparing gene methylation prevalence at baseline in the ECOG-ACRIN5597 patients who met the Medicare guidelines to receive a CT screen to screen eligible subjects from two cancer-free smoker cohorts (LSC and PLuSS) described previously [21]. In addition, the performance of our methylation panel was assessed in all ECOG-ACRIN5597 patients who provided baseline sputum compared to LSC or PLuSS current or former smokers irrespective of meeting eligibility for receiving a CT screen.

## RESULTS

### Study population

The characteristics of the entire study populations are shown in Table 1. As expected the ECOG-ACRIN LC cases were slightly older and more had quit smoking. Pack years were available for 259 LC cases and were comparable to current and former smokers in the PLuSS cohort, but significantly greater than the LSC cohort.

**Table 1:** Characteristics of study populations

Variable	ECOG-ACRIN	LSC	PLuSS	P value
N	487	1380	718	
Age (mean ± SD)	66.4 ± 8.8	57.0 ± 9.6	64.6 ± 5.1	<0.0001^2^
Sex (male, %)	268 (55)	339 (25)	234 (33)	<0.0001
Smoking status				
Current	160 (33)	756 (55)	424 (59)	<0.0001
Former	327 (67)	624 (45)	294 (41)	
Pack-years^1^ (mean ±SD)	56 ± 37	41 ± 20	55 ± 21	<0.0001^2^

^1^Pack-years were available for 259 of the ECOG-ACRIN patients.

^2^Comparing ECOG-ACRIN to LSC. P values are calculated using χ^2^ for binary or categorical variables and one-way ANOVA for continuous variables.

### Gene methylation in sputum as a classifier for lung cancer risk in CT screen eligible smokers

**Figure 1:**
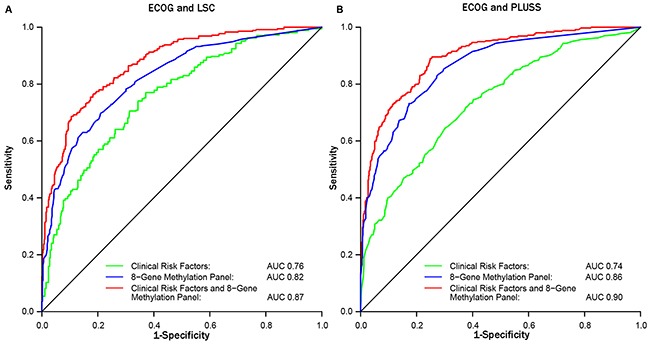
ROC curves for comparing the sensitivity and specificity for the eight-gene methylation panel with and without clinical risk factors between ECOG-ACRIN and LSC (A) or PLuSS (B) for classifying lung cancer risk

The utility of the eight gene panel to classify risk for LC was evaluated by comparing gene methylation prevalence at baseline in the 371 ECOG-ACRIN5597 patients who met the Medicare guidelines to receive a CT screen to screen eligible subjects from two cancer-free smoker cohorts (LSC [n = 466] and PLuSS [n =597]) described previously [21]. Comparative characteristics of “screen eligible” subjects are detailed in Table 2. Two analyses were performed to evaluate prediction accuracy for LC by comparing ECOG-ACRIN5597 and LSC versus ECOG-ACRIN5597 and PLuSS cohort. The prevalence for methylation of all genes was significantly increased in the ECOG-ACRIN patients compared to cancer-free smokers as evident by elevated odds ratios that ranged from 1.6 to 8.9 (Table 3). ROC curves comparing the eight-gene methylation panel for ECOG-ACRIN5597 to LSC or PLuSS showed classification accuracy of 82% and 86% (Figure 1A, 1B). ROC curves restricted to the subset of ECOG-ACRIN5597 subjects (n = 194) with pack years available were identical to those in Figure 1 (classification accuracies of 89% and 91%, respectively). Most important, the gene panel when added to the clinical variables increased the prediction accuracy from 76% to 87% (p = 7.2 × 10^−9^ for delta area under the curve [AUC]) and 74% to 90% (p =3.2 × 10^−16^ for delta AUC) when ECOG-ACRIN5597 subjects were compared to LSC or PLuSS, respectively (Figure 1, Table 4).

**Table 2:** Comparative demographics between ECOG-ACRIN, LSC, and PLuSS cohort members eligible for CT screening

Variable	ECOG-ACRIN	LSC	PLuSS	P value
N	371	466	597	
Age (mean ± SD)	67 ± 5.9	63.5 ± 5.7	64.4 ± 4.5	<0.0001
Sex (male, %)	210 (57)	119 (26)	210 (35)	<0.0001
Smoking status				<0.0001
Current	119 (32)	277 (59)	390 (65)	
Former	252 (68)	189 (41)	207 (35)	
Pack-years^1^ (mean ±SD)	67 ± 30	56 ± 24	59 ± 19	

^1^Pack-years were available for 194 of the ECOG-ACRIN patients.

P values are calculated using χ^2^ for binary or categorical variables and one-way ANOVA for continuous variables.

**Table 3:** Comparison of gene promoter methylation prevalence in sputum from ECOG-ACRIN lung cancer patients to the lovelace smokers cohort (LSC) and pittsburgh PLuSS cohort

Gene	ECOG-ACRIN (n=371)^1^	LSC (n=466)^1^	PLuSS (n=597)^1^	OR^2^ (95% CI) (ECOG/LSC)	OR^2^ (95% CI) (ECOG/PLuSS)
*P16*	132 (36)	92 (20)	89 (15)	2.3 (1.6 – 3.2)	3.2 (2.3 – 4.5)
*MGMT*	152 (42)	125 (26)	140 (23)	2.0 (1.5 – 2.8)	2.4 (1.8 – 3.2)
*DAPK*	157 (43)	87 (19)	77 (13)	3.3 (2.3 – 4.6)	5.0 (3.6 – 7.1)
*RASSF1A*	37 (10)	2 (0.4)	0	7.4 (1.8 – 31.4)	NC
*GATA4*	265 (73)	174 (37)	198 (33)	3.9 (2.8 – 5.3)	4.9 (3.6 – 6.6)
*GATA5*	164 (45)	62 (13)	55 (9)	5.3 (3.6 – 7.6)	7.9 (5.5 – 11.5)
*PAX5α*	116 (32)	87 (19)	68 (11)	1.6 (1.2 – 2.3)	3.2 (2.2 – 4.5)
*PAX5β*	117 (32)	33 (7)	30 (5)	6.2 (4.0 – 9.8)	8.9 (4.7 – 14.1)

NC, not able to calculate. ^1^ Number in parenthesis under columns of ECOG-ACRIN, LSC, and PLuSS is prevalence of gene methylation. ^2^Adjustment for age, sex, and smoking status was included in logistic regression. Cohort identifier was coded as two dummy variables with ECOG-ACRIN set as the reference.

**Table 4:** Performance of gene methylation as a classifier for lung cancer risk

	AUC (95% CI)	P value^1^	Sensitivity^2^	Specificity	PPV (%)	NPV (%)
ECOG-ACRIN vs. LSC						
Clinical risk factors	0.76 (0.73–0.79)		95	29	51	88
Methylation panel	0.82 (0.79–0.85)	0.0018	95	31	51	89
Clinical + methylation	0.87 (0.85–0.90)	7.2 × 10^−9^	95	52	60	93
ECOG-ACRIN vs. PLuSS						
Clinical risk factors	0.74 (0.71–0.79)		95	24	44	89
Methylation panel	0.86 (0.84–0.89)	1.2 × 10^−9^	95	47	52	94
Clinical + methylation	0.90 (0.88–0.92)	3.2 × 10^−16^	95	56	56	94

^1^P value for delta AUC with model containing clinical risk factors only as the reference. ^2^Sensitivity set to 95%.

Random sampling to match for the difference in distributions of age, sex, and smoking status between ECOG-ACRIN5597 and LSC/PLuSS had no effect on prediction accuracy of the gene panel (Table 4, Supplementary Figure 1). With the sensitivity set at 95%, the addition of the methylation biomarkers increased specificity from 25% (clinical variables only) to 54%, while NPV and PPV were increased from 88% to 94% and 47% to 58%, respectively (average values comparing ECOG-ACRIN5597 versus LSC/PLuSS; Table 4).

### Gene methylation classifier extends lung cancer risk assessment beyond medicare screening guidelines

The performance of the gene methylation panel was also evaluated in the ECOG-ACRIN versus LSC or PLuSS cohorts independent of their age, smoking history and smoking status (years quit), albeit everyone was 40 years and older and had smoked a minimum of 10 pack years. This design increased the samples sizes to 487, 1380, and 718 for the ECOG-ACRIN, LSC, and PLuSS cohorts, respectively. ROC curves comparing the eight-gene methylation panel for ECOG-ACRIN5597 to LSC or PLuSS each showed classification accuracy of 88% when combining clinical risk factors with the 8-gene methylation panel. Accordingly, this relaxed inclusion criteria also did not significantly diminish specificity when sensitivity was set at 95%.

## DISCUSSION

This large cross sectional study of smokers provides compelling support that a significant increase in classification accuracy and accompanied specificity for predicting LC risk can be achieved by addition of a gene methylation panel in sputum to the inclusion variables for CT screening when comparing these cancer patients to two geographically distinct cancer-free smoker cohorts. Moreover, classification accuracy of the methylation panel was similar when relaxing the Medicare inclusion criteria for CT to include all ECOG-ACRIN cases that smoked compared to all cancer-free subjects from LSC and PLuSS. A limitation of these studies was that our assessment was restricted to the ∼70% of smokers who produce sputum. However, with the advent of the Lung Flute, most individuals that do not spontaneously produce sputum will be able to provide a specimen for risk assessment [22]. Thus, implementation of this gene methylation panel for population-based screening could be a paradigm shift for LC management by providing a much improved risk assessment model that will save more lives through increased number of screen-detected cancer, while greatly reducing the number of false screens through exclusion of lower risk smokers.

The retrospective nature of this study design allowed us to define the classification accuracy of the biomarker panel in a sample size of cases (n = 487) that was comparable to that detected by the NLST screening trial of 53,439 smokers [7]. Importantly, using participants from the ECOG-ACRIN5597 trial also addressed for the first time the generalizability of a gene methylation biomarker panel for risk assessment through studying LC cases from across the U.S. and Canada with comparison to two geographically distinct cohorts of smokers. Another major distinguishing feature of our study beyond sample size from other sputum-based risk assessment publications is the continued reproducibility regarding the performance of genes within this biomarker panel across five independent studies [11, 12, 23–25]. This outcome likely results from the fact that the genes studied are not methylated in normal cells of any lineage thereby being cancer-specific and the use of the nested, MSP assay that has a reproducible sensitivity of 1 methylated allele in 20,000 unmethylated alleles to allow interrogation of sputum, a heterogeneous mixture of cells where the epithelial fraction is often less than 3% [11]. Moreover, high specificity is maintained in the stage 2 PCR for detecting methylated alleles through the use of annealing temperatures that exceed the melting point of the primers and short denaturation and extension cycles (15–20 sec; [11]). Finally, the fact that high classification accuracy was achieved through comparison of sputum from resected LC cases to controls strongly substantiates that the expanding field of injury with concomitant methylation is the major feature distinguishing cases from controls.

While our studies with a validated gene methylation panel in sputum have improved classification accuracy for LC in screen-eligible smokers as evident by an increase in specificity from 24% to 56% with sensitivity set at 95%, adding other methylated genes to our panel is unlikely to yield significant improvement due to the correlation among genes for differentiating case status [12]. Rather, independent sets of biomarkers that can be used in conjunction with this gene methylation panel are needed to significantly extend specificity. Changes in circulating metabolites that can be quantitated are emerging as sensitive readouts for many diseases and a plasma metabolome signature, because of its dimensionality resulting from genetic and epigenetic changes driving the expansion of field cancerization in the smoker's lung, could extend our prediction model beyond methylation biomarkers [26–31]. While this approach remains untested, promising recent metabolomic profiling studies of moderate sample size are identifying discriminatory metabolites with LC classification accuracy of 77–88% [32, 33].

The ultimate translation of this work should be to provide primary care and/or pulmonary physicians with the option of ordering a low cost (≤ $200) insurance reimbursable validated LC risk assessment test to guide decision making regarding receiving a CT scan. Our model to date significantly improves classification accuracy beyond the current Medicare guideline, will allow expanding the number of smokers considered for screening, and should better define eligibility for receiving a CT scan by removing smokers with a low probability for LC based on the addition of methylation to the risk assessment. Our patented technology [34] is amenable to a CLIA setting through development of robotic/liquid handling for sputum processing, DNA isolation, bisulfite modification, and assembling of the Stage I and II MSP reactions in a 96-well format in conjunction with low cost SYBR-Green based detection of methylated products using real-time PCR.

## METHODS

### Subject recruitment and biospecimen collection

Study participants were resected LC patients from a prevention trial and subjects from two geographically distinct cancer free smoker cohorts. Eligibility criteria for participation in the prevention trial included the following: age ≥ 18 years; 6 to 36 months from complete resection of histologically proven stage IA (pT1N0) or stage IB (pT2N0) non-small cell LC (carcinoid tumors were excluded [19]). The institutional review board for human studies approved the protocols and written consent was obtained from subjects. Following consent onto the correlative study, the Lovelace study coordinator sent a collection kit to the study site. Sputum was collected at time of entry onto study. Sputum was collected from 85% of ECOG-ACRIN patients within 18 months post-surgery.

Each participant was asked to provide two consecutive spontaneous sputum samples collected at home at each time point as described previously [11]. Study participants placed the sputum cups in a postage-paid mailer addressed to the study coordinator at Lovelace. Material from the second 3-day pooled sputum was used for this study. The collection of two sputum samples at each time point was based on the finding by Kennedy *et al*. [35] that the second sample has a higher success rate (80%) in producing an adequate sputum sample based on established cytologic standards, attributed to a ‘learning effect’ in adequate sputum collection. Following receipt, the sputum samples were pelleted and washed in Saccomanno's fixative. A small portion was smeared onto two or three slides and stained with Papanicoleau prior to cytologic diagnosis with the remaining sample stored at −80°C until time for DNA isolation. Sputum containing epithelial cells from the upper or lower airways has proven satisfactory for methylation assays and using these criteria, virtually 100% of samples were adequate for study [11].

Two cancer-free cohorts, the LSC and Pittsburgh PLuSS Cohort (PLuSS), were used to validate the classification accuracy of the gene methylation panel for predicting LC risk [21, 36]. These participants were cancer-free and methylation was assessed in sputum collected at cohort enrollment.

### DNA isolation and methylation specific PCR

Sputum DNA was isolated using methods previously described with yields of DNA that ranged from 5–100 μg [11, 12]. The eight genes selected were based on positive performance in our initial nested, case-control study in a Colorado cohort [11]. These genes included P16, MGMT, DAPK, RASSF1A, GATA4, GATA5, PAX5α and PAX5β. These genes are cancer specific genes methylated solely in epithelial cells. DNA was bisulfite modified and two-stage, nested methylation specific polymerase chain reaction (MSP) assays were used for increased sensitivity for detection of promoter methylation in sputum and plasma as described [11]. Methylation was scored as positive or negative based on the detection of a visible band in the gel. The immense cellular heterogeneity in sputum, where the epithelial fraction is typically <3% of the specimen, limits the ability to quantitate methylation, thus methylation was scored as positive or negative.

### Statistical analysis

The association between methylation of each gene measured in sputum collected at baseline and risk for LC using ever smokers enrolled in LSC (n=466), PLuSS (n=597), and ECOG-ACRIN5597 (n=371) was assessed using logistic regression. Study subjects were restricted to those who met the Medicare screening criteria with exception of pack years that was available for 194 of the 371 ECOG-ACRIN subjects [2]. Area under the curve (AUC) of a receiver operating characteristic (ROC) curve was calculated to assess the prediction performance of the logistic regressions with different sets of covariates. The basic model included age (as a continuous variable), sex, and smoking status (as a binary variable) that represent risk factors for LC available from all groups. Methylation status of each gene was defined as methylated or unmethylated based on the gel image with respect to detecting a methylated PCR product. The methylation status of the eight genes as eight independent variables was included in the basic model to evaluate the delta change in AUC. The methylation index approach showed prediction performance that was inferior to using the methylation status of each individual gene in the model (not shown). This may be due to the fact that individual gene methylation is low to moderately correlated between each other and their likely difference in magnitude with respect to driving lung cancer development does not support using equal weight as done with the methylation index. Estimates of sensitivity, specificity, negative and positive predictive value (NPV, PPV) were calculated. Analyses were expanded to assess AUC and ROC using all ECOG-ACRIN subjects (n = 487) compared to LSC (n = 1380) or PLuSS (n = 718) who had provided baseline sputum for methylation interrogation, irrespective of meeting Medicare screening criteria. All statistical analyses used two-sided tests and were conducted using SAS 9.3 and R 3.1.

## SUPPLEMENTARY MATERIALS FIGURE



## Abbreviations

LC: lung cancer
NLST: National Lung Screening trial
CMS: The Centers for Medicare and Medicaid
LSC: Lovelace Smokers Cohort
PLuSS: Pittsburgh PLuSS Cohort
AUC: area under the curve
ROC: receiver operator characteristic
NPV: negative predictive value
PPV: positive predictive value
