# Supplementary material for: Gene methylation biomarkers in sputum as a classifier for lung cancer risk
Source: Oncotarget. 2017 Jul 15;8(38):63978–85. doi: 10.18632/oncotarget.19255 (PMC5609978; doi:10.18632/oncotarget.19255)
Supplement: Supplementary file 1 [file oncotarget-08-63978-s001.pdf]

Gene methylation biomarkers in sputum as a classifier for lung cancer risk

SUPPLEMENTARY MATERIALS

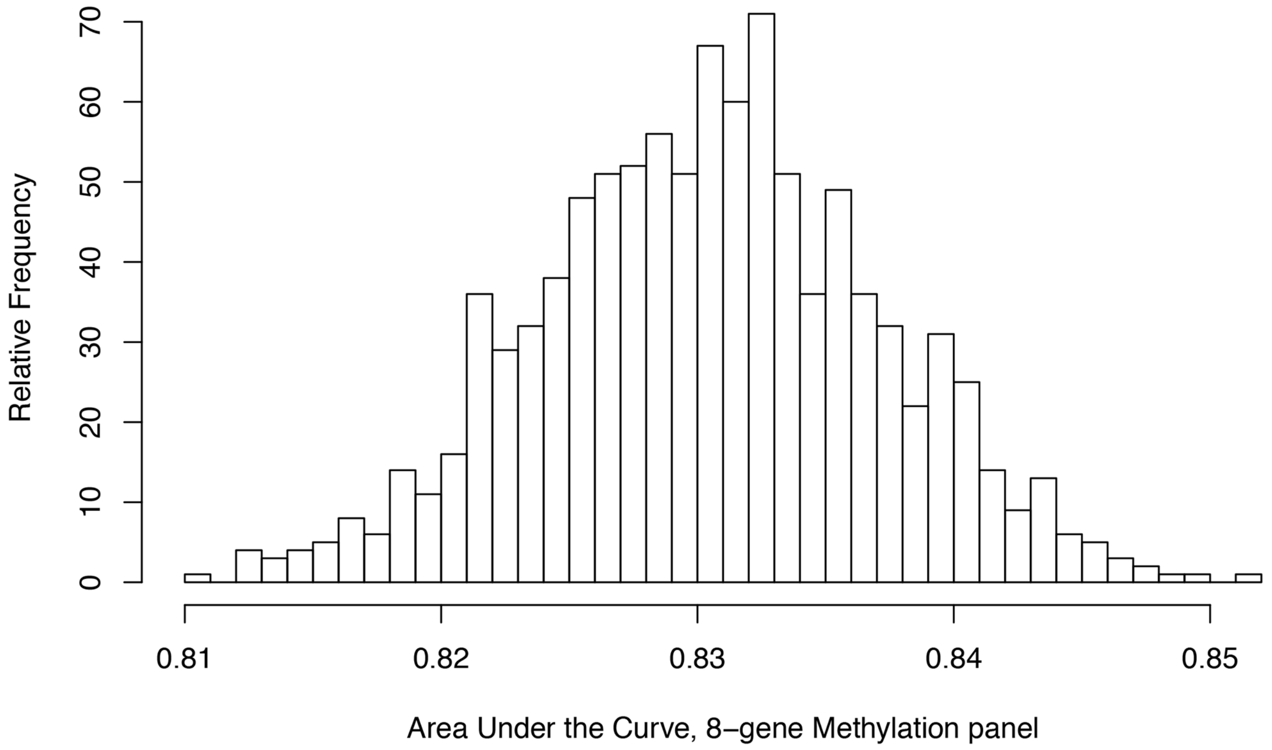

Supplementary Figure 1: Distribution of the AUC observed following subsampling from ECOG-ACRIN versus LSC/PLuSS for the model using the 8-gene methylation panel. The ECOG-ACRIN study population was categorized into four age groups representing the 25%, 50% and 75% quartile (63, 67, 72 years old, respectively). Then the same number of individuals was randomly sampled in the combined LSC and PLuSS study populations to match the sex, smoking status, and age categories in the ECOG-ACRIN cohort. In total, 1,000 iterations of sub-sampling was performed that generated 740 individuals each time (370 individuals from ECOG-ACRIN and 370 individuals out of LSC and PLuSS) to generate the AUC distribution.
